# Supplementary material for: Modeling RTT Syndrome by iPSC-Derived Neurons from Male and Female Patients with Heterogeneously Severe Hot-Spot MECP2 Variants
Source: Int J Mol Sci. 2022 Nov 21;23(22):14491. doi: 10.3390/ijms232214491 (PMC9697612; doi:10.3390/ijms232214491)
Supplement: Supplementary file 1 [file ijms-23-14491-s001.zip › ijms-1993316-supplementary.pdf]

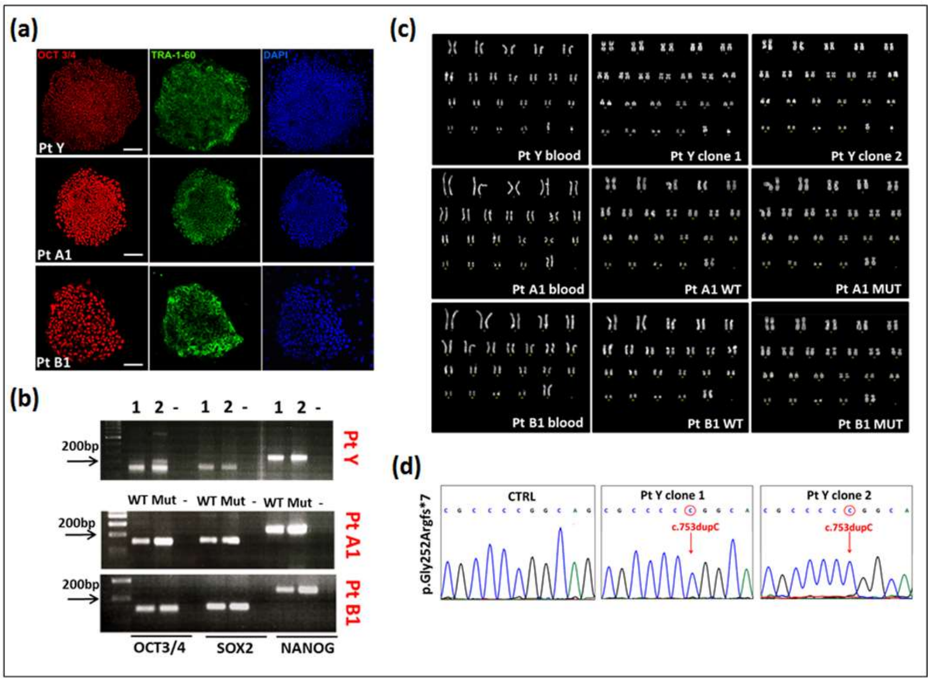

Figure S1. iPSC characterization for stemness and genome stability. Exemplification images of the characterizations in the iPSC clones: **(a)** expression of pluripotency markers by immunofluorescence analysis (IF) using antibodies against OCT3/4 (red) and TRA-1-60 (green); nuclei are counterstained with DAPI (blue): scale bar 100μm; **(b)** expression of endogenous pluripotent genes OCT3/4 (144bp), SOX2 (151bp) and NANOG (244bp) in the two independent iPSC clones derived from the male patient (PY) and the isogenic wild type (WT) and mutant (Mut) clones from PtA1 and PtB1; (**-**) no template control; **(c)** comparison between karyotype of blood and karyotype of iPSC clones of PtY (top raw), PtA1 WT and PtA1 Mut (middle raw), and PtB1 WT and PtB1 Mut (bottom raw) by scoring >20 metaphases of 2 iPSC clones confirming maintenance of the donor blood karyotype; **(d)** Sanger sequencing of DNA from the 2 clones PtY1 and PtY2 compared to a control sequence, confirming the occurrence of *MECP2* variant in the iPSC clones.

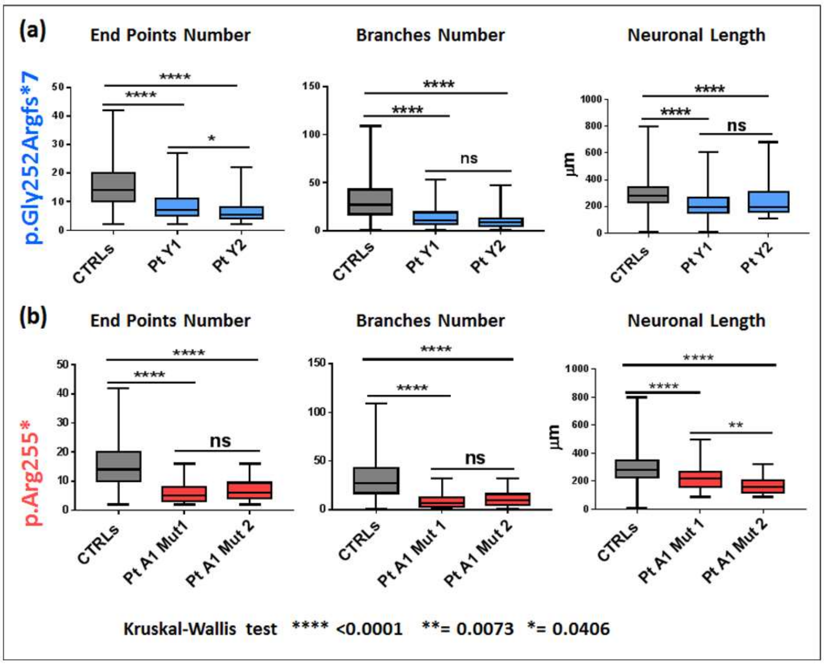

Figure S2. Morphological parameters in clones from the same patient at a comparison. (a) comparison of measures of end points number, branches number and neuronal length between each clone from the male case p.Gly252Argfs*7 with the pool of controls (CTRLs) and between the two clones (PtY1 and PtY2); **(b)** comparison of measures of end point number, branch number and neuronal length between each mutant clone from the PtA1 female p.Arg255* with the pool of controls and between the two clones (PtA1 Mut1 and PtA1 Mut2).

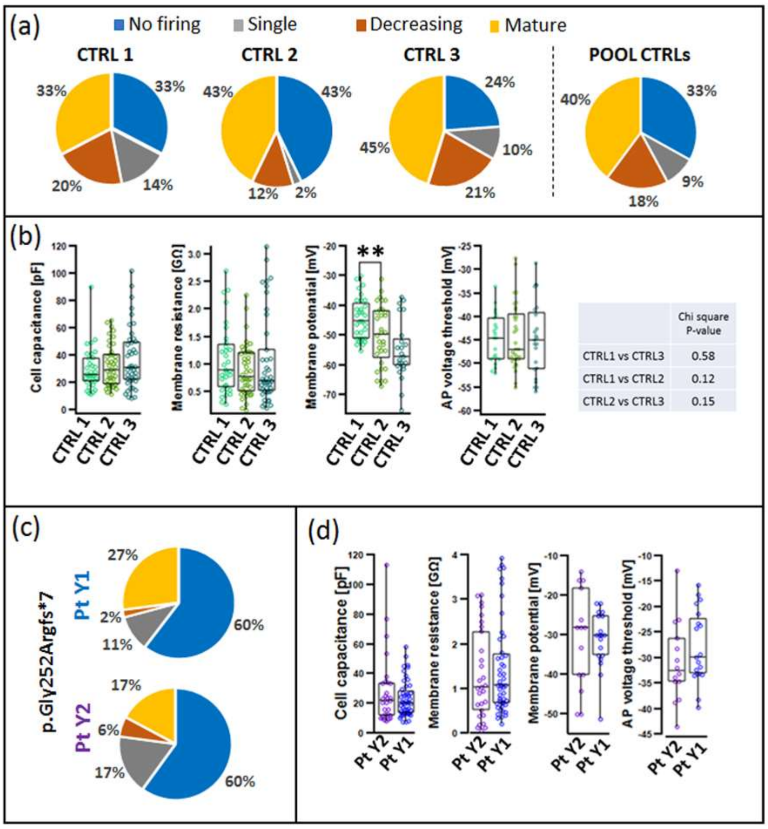

Figure S3. Functional properties of healthy control clones and of Gly252Argfs*7 male clones. Panels **(a)** and **(b)** reports the data obtained in the three clones used to generate the CTRL pool group. (a) shows the distribution of the neuron population for each CTRL group. The distributions were not statistically different when compared by chi-square test (CTRL1 vs CTRL3 $p < 0.57$, CTRL1 vs CTRL2 $p < 0.12$, CTRL2 vs CTRL3 $p = 0.15$). (b) shows the intrinsic membrane parameters for the three CTRL groups. Data were not parametric and they were represented by scatter plot on box plot which represent the 90, 75, 50, 25, and 10% of the distribution (Chi square test $p > 0.05$). In **(c)** and **(d)** we show the data of two different clones derived from the same patient bringing the mutation Gly252Argfs*7. No statistical difference was present in the firing frequency (c, chi-square $p = 0.51$) and the intrinsic properties (d, Mann–Whitney $p > 0.05$) of these groups.

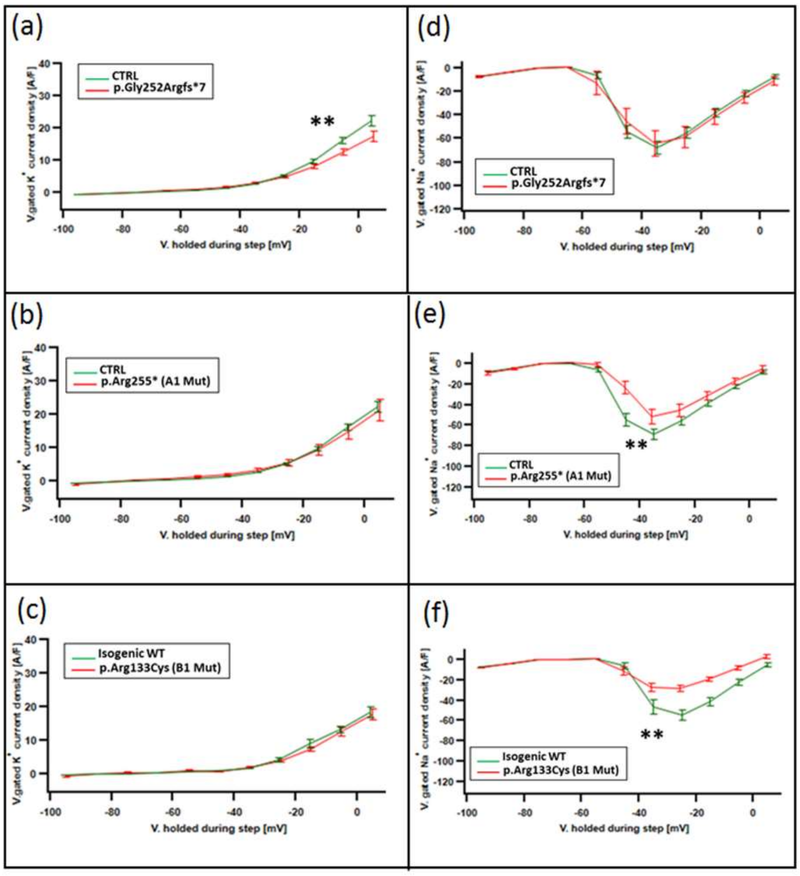

Figure S4. Voltage-gated K^+ and Na^+ currents in mutant cells. Panels (d), (e), and (f) represent the Na^+ current–voltage K^+ voltage gated current recorded in response to incremental V-steps. Panels (d), (e), and (f) represent the Na^+ curve recorded in whole cell configuration in cells with the mutations indicated in figure. (a), (b), and (c) show the K^+ voltage gated current recorded in response to incremental V-steps. Panels (d), (e), and (f) represent the Na^+ voltage gated current recorded for the same condition. For potassium, the current is measured in steady state condition at the end of the voltage step. For sodium, the data represent the maximum of the negative peak recorded after the application of voltage step. All data are reported as mean and standard error, the * means that the two distributions are different when compared with Two Factor ANOVA via Regression by a p value < 0.05 or p < 0.001 if **.
